# Complete Protection in Macaques Conferred by Purified Inactivated Zika Vaccine: Defining a Correlate of Protection

**DOI:** 10.1038/s41598-020-60415-6

**Published:** 2020-02-26

**Authors:** Ginger Young, Kelly J. Bohning, Melissa Zahralban-Steele, Greg Hather, Sambasivarao Tadepalli, Kristen Mickey, C. Steven Godin, Srisowmya Sanisetty, Stephanie Sonnberg, Hetal K. Patel, Hansi J. Dean

**Affiliations:** 1 Takeda Vaccines, Inc., Cambridge, MA USA; 2 Takeda Pharmaceuticals, Inc., Cambridge, MA USA; 3 Charles River Laboratories, Mattawan, MI USA; 4 Viracor Eurofins Lee’s Summit, Missouri, MO USA; 5 Inotiv, Gaithersburg, MD USA

**Keywords:** Infectious diseases, Vaccines, Inactivated vaccines

## Abstract

A critical global health need exists for a Zika vaccine capable of mitigating the effects of future Zika epidemics. In this study we evaluated the antibody responses and efficacy of an aluminum hydroxide adjuvanted purified inactivated Zika vaccine (PIZV) against challenge with Zika virus (ZIKV) strain PRVABC59. Indian rhesus macaques received two doses of PIZV at varying concentrations ranging from 0.016 µg − 10 µg and were subsequently challenged with ZIKV six weeks or one year following the second immunization. PIZV induced a dose-dependent immune response that was boosted by a second immunization. Complete protection against ZIKV infection was achieved with the higher PIZV doses of 0.4 µg, 2 µg, and 10 µg at 6 weeks and  with 10 ug PIZV at  1 year following vaccination. Partial protection was achieved with the lower PIZV doses of 0.016 µg and 0.08 µg. Based on these data, a neutralizing antibody response above 3.02 log_10_ EC50 was determined as a correlate of protection in macaques. PIZV elicited a dose-dependent neutralizing antibody response which is protective for at least 1 year following vaccination.

## Introduction

In 2015 and 2016, large outbreaks of Zika virus (ZIKV) occurred in the Americas. These outbreaks were associated with clusters of congenital microencephaly and other severe neurological sequelae in infections in approximately 1 of 7 infants born to pregnant women with laboratory confirmed Zika in the US and US territories^1^. Incidence of ZIKV infections subsequently declined in most of the Americas throughout 2017 and 2018^2^. With the sporadic nature of ZIKV outbreaks and a very low incidence of symptomatic disease in both endemic and non-endemic areas, conducting phase 3 clinical efficacy trials is not feasible. Still, the risk of re-emergence and the severe consequences of infection in pregnant women demonstrate that the need for an effective Zika vaccine remains. In such circumstances, alternative regulatory strategies such as Animal Rule approval or Accelerated Approval pathway may be relevant for licensure^3^.

Non-human primate studies have contributed to the development of ZIKV vaccines by demonstrating protective efficacy and identifying biomarkers of protection against ZIKV. Results to date have supported neutralizing antibodies as an immune marker that is reasonably likely to predict clinical benefit of several ZIKV vaccines^4,5^. Indian rhesus macaques (*Macaca mulatta*) are susceptible to ZIKV infection and have been used extensively as a model to study efficacy of ZIKV vaccines and pathogenesis of multiple ZIKV isolates^6–10^. ZIKV infection can be performed by subcutaneous injection, which mimics infection via mosquito bite and causes consistent viremia^11–15^. The kinetics of ZIKV infection are similar in rhesus macaques and humans where serum or plasma viremia typically peaks within the first six days of infection and resolves within 10–14 days^10,13,14^.

The purified inactivated Zika vaccine (PIZV) has previously been evaluated in mouse models and was immunogenic in AG129 and CD1 mice and protected AG129 mice against lethal ZIKV challenge^16^. In those studies, Baldwin *et al*. demonstrated that neutralizing antibodies correlate with protection in AG129 mice. PIZV is currently being evaluated for safety and immunogenicity in phase 1 trials (ClinicalTrials.gov NCT03343626).

To further evaluate immunogenicity and efficacy of PIZV, we conducted three ZIKV challenge studies in rhesus macaques. In the first study, we established a dose of PRVABC59 challenge virus. In the second study, we determined the immunogenicity and efficacy of a wide range of PIZV dose levels at 42 days after two PIZV vaccinations, to establish a potential antibody correlate of protection. In the third study, we assessed the persistence of immunity and efficacy 1 year following administration of the second PIZV dose, to evaluate neutralizing antibody kinetics and long-term protection.

## Results

### Challenge dose selection

We conducted a challenge study to select a ZIKV challenge dose that appropriately mimics human infection in Indian rhesus macaques. Macaques were challenged via subcutaneous injection with 0.5 mL containing either 10^4^ focus forming units (ffu; n = 2) or 10^5^ ffu (n = 2) ZIKV PRVABC59. Serum was collected daily for ZIKV RNA analysis by real-time quantitative RT-PCR (RT-qPCR), on days 1–11 post-infection (dpi), and every other day from day 13–21 dpi. Zika viral RNA (vRNA) was detected above the assay lower limit of quantitation (LLOQ) between days 3–8 in the 10^4^ ffu dose group and between days 2–6 in the 10^5^ ffu challenge dose group (Table 1). Macaques receiving the 10^4^ ffu challenge dose had peak vRNA of 4.9 and 5.6 log_10_ copies/mL on days 4 and 5, while macaques in the 10^5^ ffu group had peak vRNA of 4.8 and 5.0 log_10_ copies/mL, both on day 4. Zika vRNA was not detected above the assay LLOQ in the 10^4^ ffu challenge dose group after day 8 or in the 10^5^ ffu challenge dose group after day 6. The 10^4^ ffu challenge dose was selected as it resulted in higher peak vRNA and longer duration of vRNA detection than the 10^5^ ffu challenge dose.

**Table 1:** Serum vRNA per challenge dose and day post-challenge.

Challenge Dose (ffu)	Zika vRNA (log_10_ copies/mL)							
	Day 1	Day 2	Day 3	Day 4	Day 5	Day 6	Day 7	Day 8
10^4^	UD	<LLOQ	4.5	4.9	4.8	4.6	3.5	2.9
	UD	UD	4.6	4.9	5.6	5.4	UD	<LLOQ
10^5^	UD	3.3	4.6	4.8	4.3	3.8	UD	UD
	UD	3.8	4.3	5.0	5.0	4.0	UD	UD

Zika vRNA (log_10_ copies/mL) per day post-challenge for each individual macaque. Zika vRNA for individual macaques was not quantified in the 10^4^ ffu dose after day 8 or the 10^5^ ffu challenge dose group after day 6 post-challenge. UD = Undetected. <LLOQ = detected vRNA was below the assay lower limit of quantitation.

### PIZV elicits a dose dependent neutralizing antibody response

**Figure 1:**
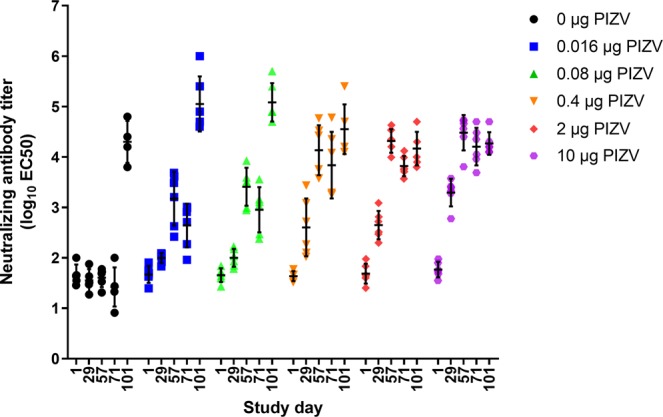
Neutralizing antibody titers following PIZV vaccination and ZIKV challenge. Rhesus macaques were vaccinated on days 1 and 29 and challenged on day 71. Individual Zika RVP titers for each vaccine group on study day 1 (prior to vaccination), day 29 (28 days post-first dose), day 57 (28 days post-second dose), day 71 (prior to ZIKV challenge), and day 101 (30 days post-ZIKV challenge). Mean and standard deviation are shown for all groups and time points.

Rhesus macaques were vaccinated with PIZV on days 1 and 29 and challenged on day 71. Serum neutralizing antibodies were tested using a Zika reporter virus particle (RVP) assay. All macaques were seronegative on day 1 and control macaques remained seronegative prior to ZIKV challenge (Fig. 1). Twenty-eight days following the first vaccine dose (study day 29), little or no seroconversion was observed for the 0.016 and 0.08 µg PIZV doses, while 4/6 macaques vaccinated with the 0.4 µg PIZV dose seroconverted and 6/6 macaques receiving the 2 and 10 µg PIZV dose seroconverted. Immune responses were boosted in all groups after the second dose (all p-values ≤0.001). Twenty-eight days after the second PIZV dose (study day 57), 100% of vaccinated macaques seroconverted. The magnitude of neutralizing antibody titers on day 57 was similar after the second dose of 0.4, 2, or 10 µg, with no statistically significant difference detected among these groups. The titer on day 57 was lower in the 0.016 (p = 0.003) and 0.08 µg groups (p = 0.037) compared to the 0.4 µg dose group. Titers prior to ZIKV challenge on day 71 were slightly decreased compared to day 57. Following challenge, neutralizing antibody titers increased significantly in the placebo, 0.016 and 0.08 µg groups (all p-values ≤0.004), but not in the 0.4, 2, and 10 µg groups. Neutralizing antibody levels after two PIZV doses of 0.4, 2, or 10 µg were similar to post-challenge neutralizing antibody titers in the placebo group. In conclusion, we observed a dose-dependent neutralizing antibody response to one or two doses of PIZV. After two PIZV doses, the neutralizing antibody levels reached a plateau for vaccine doses above 0.4 µg, and the level of neutralizing antibody was comparable to infection with ZIKV challenge in the placebo group. The lack of anamnestic antibody responses after challenge in the 0.4, 2, and 10 µg PIZV dose groups suggests that infection by ZIKV challenge virus may have been prevented by vaccination.

### PIZV elicits dose-dependent anti-Zika IgG responses

**Figure 2:**
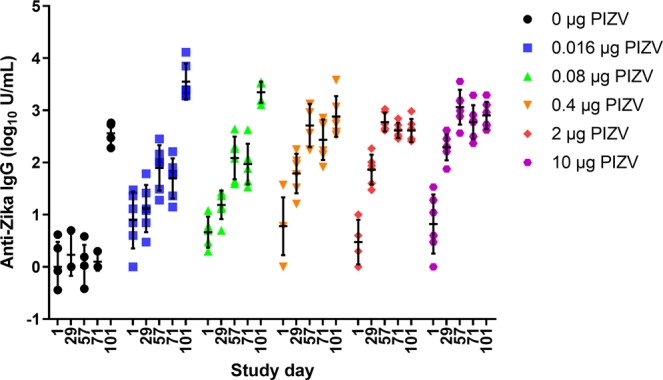
Anti-Zika IgG response following PIZV vaccination. Anti-Zika IgG concentrations for each vaccine group on study day 1 (prior to vaccination), day 29 (28 days post-first dose), day 57 (28 days post-second dose), day 71 (prior to ZIKV challenge), and day 101 (30 days post-ZIKV challenge). Mean and standard deviation are shown for all groups and time points.

In addition to evaluating neutralizing antibodies, we also quantified anti-Zika-specific IgG using a Luminex-based assay. As with neutralizing antibodies, all PIZV doses were immunogenic and no anti-Zika IgG was detected in the control group prior to ZIKV challenge. PIZV elicited dose-dependent anti-Zika IgG responses (Fig. 2) in the vaccinated groups. Anti-Zika IgG responses were significantly boosted after a second PIZV dose for all vaccinated groups (all p-values ≤0.001). Following ZIKV challenge, the anti-Zika IgG responses increased significantly in the placebo, 0.016 and 0.08 µg groups (all p-values ≤0.035), but not in the 0.4, 2, and 10 µg groups. Similar to the neutralizing antibody response, the anti-Zika IgG titers following vaccination with the 0.4, 2, or 10 µg doses were similar to post-challenge anti-Zika IgG titers in the placebo group.

### PIZV protects against ZIKV challenge

**Figure 3:**
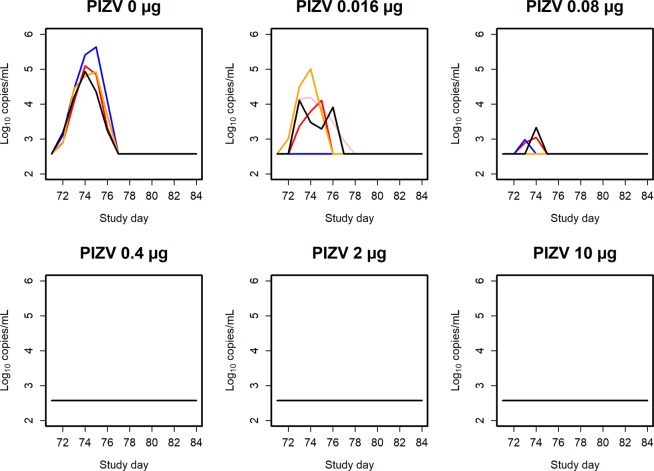
Post-challenge Zika vRNA per group and study day. Zika vRNA (log_10_ copies/mL) post-ZIKV challenge. Each line represents an individual macaque. Zika vRNA levels below the LLOQ are plotted as half of the LLOQ. Within each plot, each color represents an individual macaque.

Rhesus macaques were challenged subcutaneously on day 71 with 10^4^ ffu PRVABC59. All macaques receiving the 0.4, 2, or 10 µg PIZV dose were protected against ZIKV challenge, as no Zika vRNA could be quantified in any of the macaques in these groups. Zika vRNA was quantified in 3/6 macaques receiving the 0.08 µg dose, 4/6 macaques receiving the 0.016 µg dose, and in all macaques in the control group (Fig. 3). The peak post-challenge Zika vRNA level decreased with increasing vaccine dose level. The peak Zika vRNA occurred on day 74 (3 days post-challenge), with a geometric mean level of 5.1 log_10_ copies/mL in the control group (range 4.92–5.64 log_10_ copies/mL), 4.1 log_10_ copies/mL in the 0.016 µg dose group (range 4.10–5.00 log_10_ copies/mL) and 3.2 log_10_ copies/mL in the 0.08 µg dose group (range 2.98–3.33 log_10_ copies/mL). No clinical signs to the vaccine or challenge virus were seen throughout the studies.

### Correlate of protection

**Figure 4:**
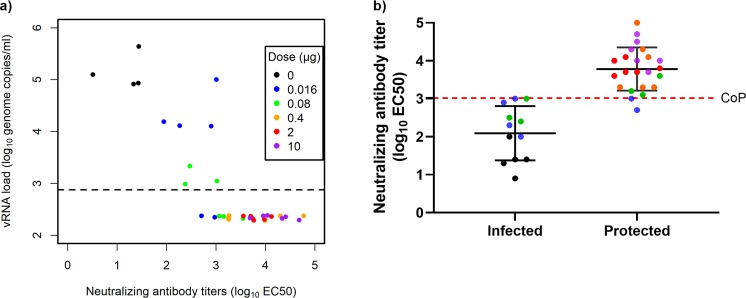
Neutralizing antibody titers negatively correlate with Zika vRNA peak value post-challenge. (**a**) Each point represents an individual macaque and respective neutralizing antibody titer (log_10_ EC50) on day of ZIKV challenge (day 71) and peak ZIKV vRNA concentration (log_10_ copies/mL) post-ZIKV challenge. For each macaque, the maximum Zika vRNA concentration observed over 10 days was plotted for analysis. Zika vRNA levels below the LLOQ are plotted as half of the LLOQ. The LLOQ is shown as a dashed line. To prevent overlaps among the points below the LLOQ, a small amount of variation has been added in the vertical direction. (**b**) To determine the correlate of protection, the neutralizing antibody titers (log_10_ EC50) of infected (positive for vRNA at any given timepoint) and protected (negative for vRNA) macaques were plotted. The correlate of protection was defined as the maximum neutralizing antibody titer across all unprotected macaques in this study. Based on this definition, the correlate of protection was established as >3.02 log_10_ EC50. CoP = Correlate of protection.

**Figure 5:**
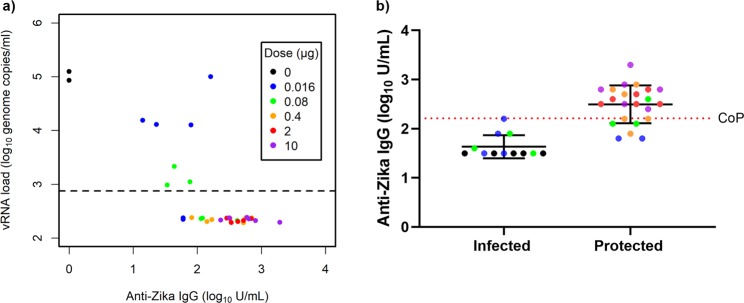
Anti-Zika IgG antibody titers negatively correlate with Zika vRNA post-challenge. (**a**) Each point represents an individual macaque. This plot demonstrates the relationship between Day 71 anti-Zika IgG and the peak vRNA concentration following ZIKV challenge. Zika vRNA values below the LLOQ were replaced by half of the LLOQ. The LLOQ is shown as a dashed line. To prevent overlaps in the plot, these data points have been jittered in the vertical direction. (**b**) To determine the correlate of protection for anti-Zika IgG, the IgG antibody titers (log_10_ U/mL) of infected (positive for vRNA at any given timepoint) and protected (negative for vRNA) macaques were plotted. Mean and standard deviation are shown for all groups and time points. The correlate of protection for anti-Zika IgG is 2.21 log_10_ U/mL and was defined as the maximum anti-Zika IgG across all unprotected macaques in this study. CoP = Correlate of protection.

PIZV elicited a dose dependent neutralizing antibody immune response and an anti-Zika IgG response which correlated with a reduction in ZIKV vRNA post-challenge (Table 2). An immune correlate analysis was subsequently performed to correlate Zika vRNA with neutralizing antibody titers (Fig. 4) and anti-Zika IgG (Fig. 5), which demonstrated that an increase in both neutralization antibody and anti-Zika IgG titers correlates with a decrease in vRNA concentration. Correlation with protection was observed with both neutralizing antibodies (3.02 log_10_ EC50) and anti-Zika IgG (2.21 log_10_ U/mL). We chose the functional assay, neutralizing antibodies, to establish a correlate of protection in macaques as a neutralizing antibody titer of >3.02 log_10_ EC50, which conferred protection against ZIKV serum viremia in Indian rhesus macaques. Due to overlap among the distributions of neutralizing antibody titers between the protected and infected animals, titers in some protected animals were below the correlate of protection.

**Table 2:** Neutralizing antibody titers on day of ZIKV challenge, Zika vRNA levels post-challenge.

PIZV dose (µg)	Mean neutralizing antibody titer (log_10_ EC50), day 71	Range of peak Zika vRNA detection (log_10_ copies/mL)	Percent macaques protected from ZIKV challenge
0	1.33	4.92–5.64	0
0.016	2.63	4.10–5.00	33
0.08	2.94	2.98–3.33	50
0.4	3.81	<LLOQ	100
2	3.81	<LLOQ	100
10	4.19	<LLOQ	100

Summary of mean neutralizing antibody titers (log_10_ EC50), range of peak vRNA post-ZIKV challenge (log_10_ copies/mL), and percent of macaques with quantifiable Zika vRNA post-ZIKV challenge. All reported and analyzed data is above the RT-qPCR LLOQ. < LLOQ = detected vRNA was below the assay lower limit of quantitation.

### Immunogenicity and efficacy 1 year post-vaccination

**Figure 6:**
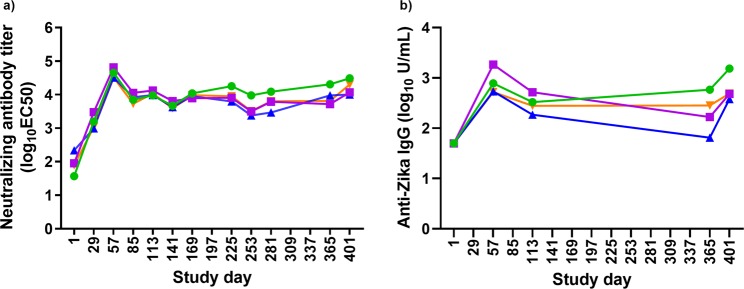
Long-term immunogenicity following PIZV vaccination. Both neutralizing antibody (**a**) and anti-Zika IgG titers were determined (**b**). Macaques were vaccinated on days 1 and 29 and challenged on day 371. Key time points include study day 1 (prior to vaccination), day 29 (28 days post-first dose), day 57 (28 days post-second dose), day 365 (prior to ZIKV challenge), and day 401 (30 days post-ZIKV challenge). Each line represents an individual macaque.

To determine the kinetics of neutralizing antibodies over a year long period, a separate set of four male Indian rhesus macaques were vaccinated with 10 µg PIZV on study days 1 and 29 and challenged with ZIKV PRVABC59 on study day 371. Zika neutralizing antibody levels were measured every 28 days through day 365 (except for days 197, 309, and 337), as well as on day 401 (30 days post-ZIKV challenge). All macaques seroconverted following the first vaccine dose, with a significant boost in antibody titers following the second dose (p < 0.001; Fig. 6a). Neutralizing antibody titers peaked on day 57, 28 days following the second immunization, declined from day 57 to day 85, and then remained stable from day 85 to day 401. Anti-Zika IgG levels also peaked on day 57 and subsequently declined through study day 365 (Fig. 6b). IgG titers increased following ZIKV challenge.

No Zika vRNA was detected in serum from any of the macaques following ZIKV challenge 1 year following the second dose. In addition, no statistically significant increase in neutralizing antibodies was observed post-ZIKV challenge on day 401 (log_10_ EC50 range of 3.72–4.31 on day 365 compared to log_10_ EC50 range of 4.00–4.49 on day 401). The combined results suggest that two doses of PIZV prevented ZIKV infection 1 year post-vaccination. In conclusion, two 10 µg PIZV vaccinations elicits persistent neutralizing antibodies and long-term protection in rhesus macaques.

## Discussion

We demonstrated that PIZV elicits a dose-dependent response of both Zika neutralizing and anti-Zika IgG antibodies. PIZV elicited neutralizing antibodies that persisted for at least 1 year and protected against ZIKV challenge. Vaccinating with a broad range of PIZV dose levels enabled us to correlate both neutralizing and anti-Zika IgG antibody titers to protection against ZIKV infection. We determined a neutralizing antibody correlate of protection of 3.02 log_10_ EC50, which we define as the maximum EC50 among the unprotected animals in the study. We chose the neutralizing antibody assay to establish a correlate of protection as the literature supports using functional neutralizing antibodies as a correlate of protection for Zika and other flaviviruses.

Neutralizing antibodies directed against the envelope (E) protein have been identified as correlates of protection for vaccines Japanese encephalitis virus (JEV), yellow fever virus, and tickborne encephalitis viruses^4^. Other laboratories using different vaccine platforms (DNA, RNA, inactivated virus, protein subunit, adenovirus-vectored, VLP) have reported induction of ZIKV-specific neutralizing antibodies that conferred protection against live ZIKV challenge in animal models^6–8,16–22^. These data support ZIKV E-specific neutralizing antibodies as a mechanism of protection against ZIKV infection.

In flavivirus vaccine development, neutralizing antibody titers that can confer protection against viremia have be reported in animal models using several different assay methods: microneutralization (MNT), RVP, and plaque reduction neutralization (PRNT). Studies of other candidate ZIKV vaccines have demonstrated that a neutralizing antibody titer as low as 100, using a MNT assay, or a titer of 1,000 using an RVP assay can confer protection against viremia in animal models^6,16,22^. These results are qualitatively similar to those of licensed vaccines against flaviviruses such as JEV, where a neutralizing antibody titer of >10 (as measured by PRNT) is considered to correlate with protection^23–25^. Further supporting this correlate of protection, it has been reported that passive transfer of Zika neutralizing monoclonal antibodies or antisera from vaccinated humans and animals to mice is sufficient to protect against ZIKV challenge^16,17,21,26^. Adoptive transfer of serum from immunized mice fully protected against viremia, while splenocytes from the same donors provided marginal protection^27^ demonstrating that the mechanism of protection against ZIKV infection is antibody mediated.

While ZIKV exists as three genotypes (West African, East African and Asian), they all behave as a single serotype^28,29^. Our previous studies demonstrated that PIZV is capable of eliciting antibodies that neutralize both African and Asian ZIKV isolates *in vitro*^16^. Others have shown that the magnitude or duration of viremia in unvaccinated macaques challenged with ZIKV isolates from Brazil or Puerto Rico is similar^6^, and that vaccinated macaques are protected against challenge with heterologous ZIKV strains^13,21,30^. Protection of animal models against heterologous challenge and cross-neutralization capabilities of antisera from multiple vaccine platforms^8,17,18,21^ suggest that data from a single challenge strain may be sufficient to show cross protection against ZIKV strains from other lineages. Several groups have developed rhesus macaque challenge models using Zika strain PRVABC59^6,7,9,12,31^ which is a well-documented and characterized isolate from human serum and is representative of viruses that were circulating in the Americas during the 2015–2016 outbreak^16^. We therefore selected PRVABC59 as the challenge strain for our studies.

In this study, we extended our observation of PIZV efficacy in mice^16^ to demonstrate efficacy in prevention of ZIKV vRNA and to evaluate anamnestic antibody responses after ZIKV challenge in non-human primates. We did not assess presence of vRNA in tissues. Our working hypothesis is that prevention of serum ZIKV vRNA may be a surrogate for prevention of the most serious sequelae of ZIKV infection in humans - fetal infection. By employing a PIZV dose titration study design and a ZIKV RVP assay, we have established a minimum protective vaccine dose of 0.4 µg in rhesus macaques and established a neutralizing antibody correlate of protection against ZIKV challenge of 3.02 log_10_ EC50. Finally, we demonstrated that ZIKV neutralizing antibodies persist and are capable of preventing ZIKV infection for at least 1 year post-vaccination. In the event that human phase 3 efficacy studies are not feasible, identifying a correlate of protection in an appropriate non-human primate challenge model may be important to support licensure of a Zika vaccine^3,5,32^. Altogether, these data support neutralizing antibodies as an immune marker that is associated with efficacy in a relevant animal model and that may predict a reasonable likelihood of clinical benefit in humans.

## Methods

### Vaccine

PIZV is an aluminum hydroxide adjuvanted whole purified inactivated virus vaccine based on ZIKV strain PRVABC59 which was originally isolated from serum from a human infected in Puerto Rico (GenBank accession number KU501215). PIZV has been previously described and characterized by Baldwin *et al*.^16^. The same lot of PIZV used in this study was also used in clinical trial ZIK101 (ClinicalTrials.gov NCT03343626).

### Rhesus macaque challenge studies

Thirty-five Indian rhesus macaques were separated into 6 groups. Three male and three female macaques per group were vaccinated intramuscularly with either 0.016, 0.08. 0.4, 2 or 10 µg PIZV on study days 1 and 29. To achieve statistical significance comparing vaccine doses, six animals/group were selected to obtain >80% power if the true infection rate among controls is ≥90% and the true infection rate among vaccinated animals is ≤16% based on a one-sided Fisher’s Exact Test with 5% Type 1 error rate^7^. Two male and three female macaques were vaccinated with placebo control (PBS) on study days 1 and 29. One female from the control group was euthanized on the day of ZIKV challenge due to reasons unrelated to the study, resulting in 4 macaques in the control group. Macaques were challenged subcutaneously with 10^4^ ffu/0.5 mL ZIKV PRVABC59 on study day 71. Serum samples were collected and tested for antibody titers on study days 1, 29, 57, and 71, and 101, and for ZIKV RNA on study days 71–80, and study day 84.

A second study was conducted to assess long-term PIZV immunogenicity and efficacy. Flavivirus seronegative male rhesus macaques (n = 4) were vaccinated with the 10 µg PIZV dose on days 1 and 29 and challenged subcutaneously with 10^4^ ffu/0.5 mL ZIKV PRVABC59 on day 371. Serum samples were collected and tested for antibody titers monthly up to 1 year post-vaccination and 30 days following ZIKV challenge (study day 401). To assess replication of Zika vRNA following ZIKV challenge, serum samples were collected on study days 371–381, and 385.

### Challenge virus

Zika virus Puerto Rico strain PRVABC59 was used for the challenge (0.5 mL containing a nominal dose of 10^4^ ffu). The seed stock was passaged three times on Vero cells at CDC, Fort Collins, Colorado. Virus stocks were prepared by passaging two times on C6/36 mosquito cells. Stocks were from a master working virus bank and tested for sterility, mycoplasma and endotoxin prior to use.

### Animals

Indian rhesus macaques were screened for antibodies against flaviviruses, Herpes B Virus, Simian Retrovirus, Simian Immunodeficiency Virus, Simian T Lymphotropic Virus, Mycobacterium Tuberculosis, Simian Varicella Virus, Malaria, Salmonella, Shigella, Yersinia and internal parasites. Macaques were housed at either Charles River Laboratories or Inotiv. All in-life practices were approved and conducted per the Institutional Animal Care and Use Committee protocols (protocol number 2715–001 and 2715–002, or 2384–14376, respectively). Animals were evaluated twice daily for clinical signs following vaccination and challenge.

### Screening

To select flavivirus naïve macaques, baseline IgG serostatus was determined using a multiplex Luminex kit (Ampersand Biosciences Flavivirus Serological Panel). Briefly, macaque serum was diluted 1:2,000 in sample diluent and added to multiplexed magnetic beads coupled with Zika, Dengue, Yellow Fever, Japanese Encephalitis, West Nile, Usutu, Saint Louis Encephalitis, and Chikungunya virus antigens. Serum and beads were incubated on a plate shaker for 1 hour at room temperature and then washed three times with assay buffer. Detection antibody, anti-IgG PE, was added to each sample and incubated on a plate shaker for 30 minutes at room temperature. Following another three wash cycles, beads were resuspended in assay buffer and read on the Bio-Plex MAGPIX to measure median fluorescent intensity (MFI) for each bead set. Macaques were considered flavivirus naïve and selected for the study if the MFI was below pre-defined assay cutoff criteria for seronegativity for all antigens.

### Neutralizing antibodies

A Zika RVP assay (Sonnberg *et al*., manuscript in preparation) was used to determine neutralizing antibody titers in serum following the administration of PIZV (study days 1, 29, 57, and 71), and 30 days post-ZIKV challenge (day 101). Briefly, macaque serum was heat inactivated at 56 °C for 30 minutes and then serially diluted 3-fold in assay media for an 11-point dilution series. Diluted serum and RVP were plated in duplicate in a 384-well assay plate and incubated for 1 hour at 37 °C. Vero cells were added to each well and incubated at 37 °C for 72 hours. Renilla-Glo substrate (Promega, WI, USA) was then added to the plate and incubated for 15 minutes at room temperature. Finally, plates were analyzed by a luminometer. The effective concentration at 50% (EC50), was determined by a non-linear regression curve fit with the lower asymptote constrained to 0 in GraphPad Prism. The LLOQ for the NHP assay is 2.12 log_10_ EC50, below which the serum matrix interfered with the measurement. The upper limit of quantitation (ULOQ) of the standard assay is 5.0 log_10_ EC50. Any samples returning titers >ULOQ were retested with a higher initial pre-dilution.

### Anti-Zika IgG binding antibodies

Anti-Zika IgG antibody levels were measured for study days 1, 29, 57, and 71 (prior to ZIKV challenge) using a Luminex based anti-Zika IgG assay. Heat-inactivated serum samples were diluted 1:100 in assay buffer and then serially diluted 4-fold for an 8-point dilution curve. Magnetic beads covalently coupled with PIZV antigen were added to each sample dilution in a 96-well plate and each sample dilution was tested in duplicate. Plates were incubated on a plate shaker for 90 minutes at room temperature and then washed two times with assay buffer. Diluted anti-Ig-PE detection antibody was then added to each sample and incubated on a plate shaker for 1 hour at room temperature. After two wash cycles, beads were resuspended in assay buffer and read on the Luminex FLEXMAP 3D to measure the MFI. The IgG antibody concentration was quantified using a reference standard serum with an assigned IgG concentration in units/mL (U/mL). The LLOQ for the assay is 1.5 log_10_ U/mL.

### RT-qPCR

Total RNA from serum samples was extracted using the Qiagen BioRobot Universal Instrument and QIAamp Virus BioRobot MDx Kit (Qiagen; Valencia, CA). Extracted and purified RNA was evaluated in two separate RT-PCR assays. Primarily, Zika vRNA was detected and quantified using RT-qPCR with primers and probe specific to known ZIKV genotypes as described in Lanciotti *et al*.^33^ and a standard curve generated from synthetic reference Zika vRNA (ATCC). A qualitative extraction control RT-PCR was also performed. The extraction control RT-PCR utilized a primer/probe set, specific to *Macaca mulatta* C1GALT1C1L mRNA, confirmed adequate nucleic acid extraction from serum samples independent of Zika vRNA detection. The geometric mean, range of peak Zika vRNA detection, and the respective percentage of protected macaques (as defined by absence of vRNA) was calculated using quantities greater than the assay lower limit of quantitation of 2.9 log_10_ copies/mL. Samples with vRNA concentration lower than the assay limit of detection of 2.3 log_10_ copies/mL were considered negative.

### Correlate of protection

The mean neutralizing antibody titers (log_10_ EC50) determined by Zika RVP assay was computed for each dose group at day 71 (day of ZIKV challenge). Zika vRNA copies/mL determined by RT-qPCR assay were computed for each dose and timepoint post-challenge (study days 71–81 and 84). Peak vRNA for each macaque was defined as the highest observed vRNA concentration across all timepoints tested. Macaques were considered protected if vRNA was not detected or was below the assay LLOQ for all timepoints tested. The correlate of protection was defined as the maximum neutralizing antibody titer across all unprotected macaques in this study. This definition was conservative in that some protected macaques could have neutralizing antibody titer levels below the correlate of protection due to overlap between the distributions of protected and unprotected macaques. Since the correlate of protection was not a statistical estimate, no confidence intervals were reported.

### Statistical analysis

Statistical tests were performed using the R software version 3.5.1.; log_10_ neutralization titers were compared for selected pairs of days for each dose group using a paired two-sided t-test. At day 57, Tukey’s test was used to compare all dose groups with each other. A two-sided Spearman’s test was applied to the unprotected macaques to check for an association between Day 71 neutralizing antibodies titers and peak vRNA. Comparisons between selected pairs of days were also performed with the log_10_ IgG antibody levels in the main study and with the log_10_ neutralization titers from the long-term immunogenicity study using paired two-sided t-tests. A p-value threshold of 0.05 was used to determine statistical significance.

## Data Availability

The data that support the findings of this study are available from the corresponding author upon reasonable request and with permission of Takeda Vaccines, Inc.
